# Human Brucellosis in Iraq: Spatiotemporal Data Analysis From 2007-2018

**DOI:** 10.2196/54611

**Published:** 2024-07-03

**Authors:** Ali Hazim Mustafa, Hanan Abdulghafoor Khaleel, Faris Lami

**Affiliations:** 1 Department of Inspection, Ministry of Health, Baghdad, Iraq; 2 Surveillance Section, Communicable Diseases Control Center, Public Health Directorate, Ministry of Health, Baghdad, Iraq; 3 College of Medicine, University of Baghdad, Baghdad, Iraq

**Keywords:** human brucellosis, livestock, clustering, spatial, temporal, Iraq

## Abstract

**Background:**

Brucellosis is both endemic and enzootic in Iraq, resulting in long-term morbidity for humans as well as economic loss. No previous study of the spatial and temporal patterns of brucellosis in Iraq was done to identify potential clustering of cases.

**Objective:**

This study aims to detect the spatial and temporal distribution of human brucellosis in Iraq and identify any changes that occurred from 2007 to 2018.

**Methods:**

A descriptive, cross-sectional study was conducted using secondary data from the Surveillance Section at the Communicable Diseases Control Center, Public Health Directorate, Ministry of Health in Iraq. The trends of cases by sex and age group from 2007 to 2018 were displayed. The seasonal distribution of the cases from 2007 to 2012 was graphed. We calculated the incidence of human brucellosis per district per year and used local Getis-Ord *G*_i_* statistics to detect the spatial distribution of the data. The data were analyzed using Microsoft Excel and GeoDa software.

**Results:**

A total of 51,508 human brucellosis cases were reported during the 12-year study period, with some missing data for age groups. Human brucellosis persisted annually in Iraq across the study period with no specific temporal clustering of cases. In contrast, spatial clustering was predominant in northern Iraq.

**Conclusions:**

There were significant differences in the geographic distribution of brucellosis. The number of cases is the highest in the north and northeast regions of the country, which has borders with nearby countries. In addition, people in these areas depend more on locally made dairy products, which can be inadequately pasteurized. Despite the lack of significant temporal clustering of cases, the highest number of cases were reported during summer and spring. Considering these patterns when allocating resources to combat this disease, determining public health priorities, and planning prevention and control strategies is important.

## Introduction

Brucellosis is one of the most widespread zoonotic diseases in the world, responsible for enormous economic losses and considerable human morbidity in endemic areas [1]. The World Health Organization estimates that there are 500,000 annual new infections in over 170 countries, and 9% of them were from the Eastern Mediterranean region [2,3].

Most (90%) human infections are subclinical, resulting in delayed diagnosis and complications [1]. Human-to-human transmission is rare, and human infection can result from direct contact with infected animals or their products, the consumption of contaminated raw milk and milk products, or inhalation [1]. The main sources of brucellosis infection are most goats and sheep (*Brucella melitensis*), cattle (*Brucella abortus*), pigs (*Brucella suis*), and dogs (*Brucella canis*) [3]. Brucellosis seasonality shows no specific pattern. However, it usually coincides with the livestock breeding season [3] when human exposure to livestock or their contaminated products occurs, leading to more human infections in locations with infected livestock [4].

A study from northern Iraq showed that the prevalence of brucellosis in livestock varied from 1% to 70%, depending on the species and diagnostic methods [4]. That is, the seroprevalence of brucellosis in studies that used the Brewer card test was 3.1% for cattle, from 0.7% to 1% for sheep, from 2.5% to 4.4% for goats, and 2% for combined sheep and goat [5]. A study that used the Rose-Bengal test found a 5.5% prevalence in sheep and 5.3% in goats [6]. Studies that used competitive enzyme-linked immunosorbent assay (ELISA) found a brucellosis prevalence of 16.7% in cattle and 50% in buffalo [7]. The varying prevalence of brucellosis in livestock can have an effect on brucellosis prevalence in humans. In addition, the evaluation of veterinary and human public health measures can build on the finding of these studies. The veterinary vaccination program started in 2007 in Iraq; however, its implementation, like the implementation of all other health services and interventions, was negatively affected by political insecurities in the region, leading to suboptimal vaccination rates [4].

Currently, no satisfactory vaccine is available for humans [8]. Brucellosis control depends on testing and isolating or slaughtering brucellosis-positive animals, vaccinating susceptible animals, and controlling animal movements [9].

In Iraq, there is no prior description of the spatiotemporal epidemiology of human brucellosis. This study uses official data from the Ministry of Health (MoH) to identify potential changes in the spatial and temporal occurrence of human brucellosis cases in Iraq from 2007 to 2018.

## Methods

### Study Design

This was a descriptive, retrospective study of the spatial and temporal distribution of human brucellosis from 2007 to 2018.

### Data Source

Human brucellosis data were extracted from the surveillance database at the Surveillance Section at the Communicable Diseases Control Center (CDC), Public Health Directorate, MoH in Iraq. The data included the number of human cases classified by the reporting districts for all 18 provinces in the country. The data for the study included information on sex, age group, the reporting district, and time of diagnosis (year). The number of human brucellosis cases in Iraq from 2007 to 2012 was retrieved from the Iraq CDC as the total number of patients at the provincial level; therefore, no further spatial analysis was done. From 2012 to 2018, the data were aggregated by province, district, age group, and sex; therefore, spatial analysis was done at the district level. Patients’ sex was grouped as female and male. Age was grouped as younger than 1, 1‐4, 5‐14, 15‐45, and older than 45 years in the aggregated form.

Up to 2018, all 18 Iraqi governorates must report human cases of brucellosis to the Surveillance Section using monthly aggregated forms by district. The presumptive diagnosis was made by the rapid brucellosis test, whereas the confirmatory test was done using ELISA or polymerase chain reaction test.

Data of each governorate’s total population in Iraq and the population distribution by age and sex were retrieved from the Central Statistical Organization, Ministry of Planning, Iraq and used to calculate the incidence for the studied years (2007‐2018). The incidence of human brucellosis was calculated as follows:

Incidence = number of new cases of human brucellosis in 1 year / district population for the same year × 100,000

No data about the population in each district of Kurdistan provinces for 2007, 2008, and 2009 were available from the Central Statistical Organization. Therefore, data from 2007 to 2012 were analyzed in graphs and tables using Excel software (2019; Microsoft). The 12-year study period was grouped into 2 parts: the first part spanned from 2007 to 2012 when the data were aggregated at the provincial level, and the second part spanned from 2013 to 2018 when the data were aggregated at the district level, to analyze and describe the spatial and temporal distribution of the disease.

### Statistical Analysis

A descriptive analysis of human brucellosis during the first period was done by calculating the cases’ frequency and percentage according to age group, sex, and season. In addition, we described the trends of sex and age group distribution from 2007 to 2018 using a stacked 100% bar chart and table to detect any changes during this period.

We used local Getis-Ord *G*_i_* and Getis-Ord *G*_*i*_ statistics to identify the local concentration of high and low values in neighboring districts and their the statistical significance. The Getis-Ord *G*_*i*_ statistic is the ratio of the weighted average of the values in the neighboring locations to the sum of all values, not including the value at the location (*x*_*i*_) [10]:


$$G_{i}=\frac{\sum_{j\neq i}w_{ij}x_{j}}{\sum_{j\neq i}x_{j}}$$

In contrast, the local Getis-Ord *G*_i_* statistic includes the value *x*_*i*_ in both the numerator and denominator:


$$G_{i}^{*}=\frac{\sum_{j}w_{ij}x_{j}}{\sum_{j}x_{j}}$$

High values of either the local Getis-Ord *G*_*i*_ or Getis-Ord *G*_i_* coefficient point to a concentration of districts with a high number of brucellosis cases, whereas low values point to a clustering of districts with a low number of cases. *P* values of .05 and lower were considered statistically significant. If the *P* value is statistically significant and the *z*-score is positive, then the spatial distribution of high and low values in the data set is more spatially clustered than would be expected if the underlying spatial processes were truly random. In contrast, if the *P* value is statistically significant and the *z*-score is negative, then the spatial distribution of high and low values in the data set is more spatially dispersed than would be expected if the underlying spatial processes were truly random. A dispersed spatial pattern often reflects some type of competitive process: a feature with a high value repels other features with high values and vice versa. *P* values of .05 and lower that were considered statistically significant were grouped at 3 thresholds (.05, .01, and .001) as the levels of significance increase with the lowest *P* value. Maps and tests were done using GeoDa software (version 1.12.1.161; September 2018; Center for Spatial Data Science at the University of Chicago).

### Ethical Considerations

Administrative and ethical approval was granted from the Public Health Directorate, MoH in Iraq (#5818).

## Results

**Figure 1.**
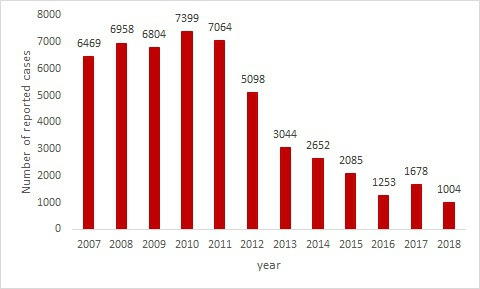
Frequency of reported human brucellosis cases in Iraq from 2007 to 2018.

The total number of human brucellosis cases reported in Iraq from 2007 to 2018 was 51,508. The disease trend showed 2 peaks, one in 2010 and another in 2011, with 7399 and 7064 recorded cases, respectively. There was an apparent decline in reported cases from 2013 to 2018, as shown in Figure 1.

**Figure 2.**
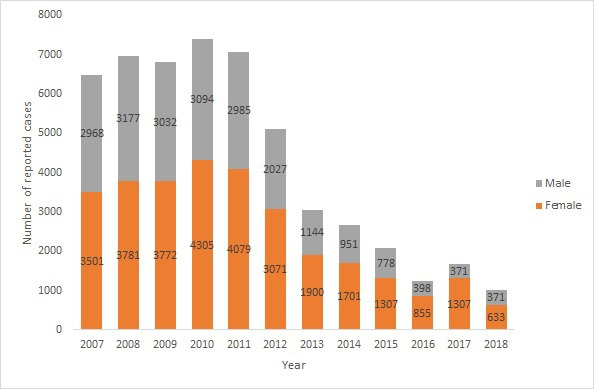
Sex distribution of human brucellosis cases in Iraq from 2007 to 2018.

Most cases were female (30,212/51,508, 58.02%), with increased frequency in 2016 and 2017, followed by decline in 2018 (Figure 2). Overall, 61.19% (30,977/50,621) of the cases were aged 15‐45 years, with no apparent change in the age groups affected throughout the years (some data were missing for age groups; Table 1).

**Table 1.** Age group distribution of human brucellosis cases in Iraq from 2007 to 2018. Some data were missing for age groups.

Age group (y)	Year and cases, n (%)											
	2007 (n=6492)	2008 (n=6958)	2009 (n=6804)	2010 (n=7399)	2011 (n=7061)	2012 (n=5067)	2013 (n=3069)	2014 (n=2643)	2015 (n=2081)	2016 (n=1252)	2017 (n=1004)	2018 (n=791)
>45	1356 (20.89)	1788 (25.7)	1778 (26.13)	1608 (21.73)	1650 (23.37)	1080 (21.31)	679 (22.12)	607 (22.97)	421 (20.23)	252 (20.13)	210 (20.92)	165 (20.86)
15‐45	3868 (59.58)	3905 (56.12)	3937 (57.86)	4744 (64.12)	4250 (60.19)	3092 (61.02)	1993 (64.94)	1698 (64.25)	1445 (69.44)	879 (70.21)	668 (66.53)	498 (62.96)
5‐14	1099 (16.93)	1148 (16.5)	931 (13.68)	938 (12.68)	1000 (14.16)	766 (15.12)	358 (11.67)	280 (10.59)	193 (9.27)	111 (8.87)	110 (10.96)	113 (14.29)
1‐4	159 (2.45)	108 (1.55)	150 (2.2)	92 (1.24)	148 (2.1)	112 (2.21)	36 (1.17)	55 (2.08)	20 (0.96)	8 (0.64)	14 (1.39)	14 (1.77)
<1	10 (0.15)	9 (0.13)	8 (0.12)	17 (0.23)	13 (0.18)	17 (0.34)	3 (0.1)	3 (0.11)	2 (0.1)	2 (0.16)	2 (0.2)	1 (0.13)

**Figure 3.**
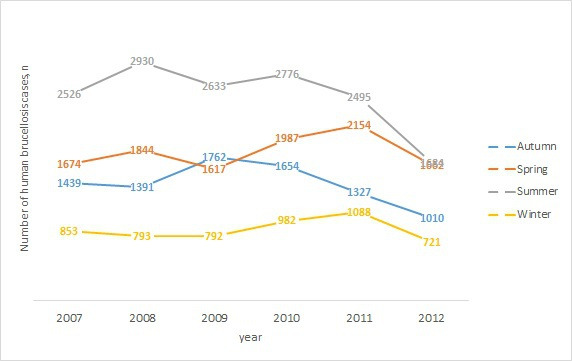
Seasonal distribution of human brucellosis cases in Iraq from 2007 to 2012.

Seasonal distribution of human brucellosis cases was constant throughout the year (Figure 3). The cases occurred the most frequently in summer (15,044/39,794, 37.8%), followed by spring (10,938/39,794, 27.49%).

**Figure 4.**
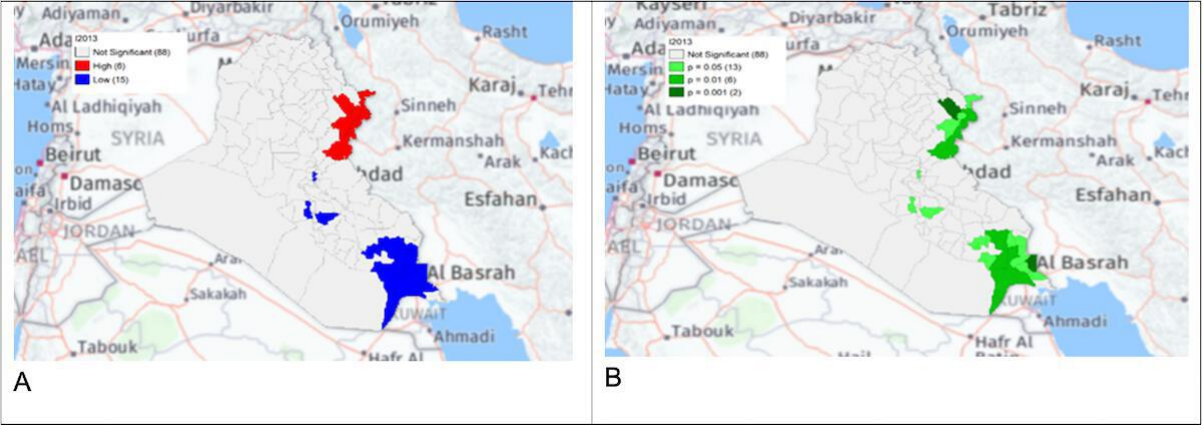
Gi* Cluster (A) and Gi* Significance (B) map of human brucellosis cases in Iraq in 2013.

The local Getis-Ord *G*_i_* statistics showed increased hot spots (high-high clusters) and cold spots (low-low clusters) from 2013 to 2018. Hot spots were located in the north and northeastern parts of Iraq, that is, in districts Choman, Soran, and Erbil in Erbil province; district Raniya in Sulaymaniyah province; district Akre in Nineveh province; districts Kirkuk and Dibis in Kirkuk province; districts Alshirqat, Baiji, and Tikrit in Salah Al-Din province; and district Amedi in Dahuk province. In contrast, cold spots were located in districts located in the provinces of Thiqar, Muthanna, Maysan, Anbar, Najaf, and Baghdad. District Dibis in Kirkuk shifted from a cold spot in 2015 to a hot spot in 2016. District Koisanjaq in Erbil shifted from a hot spot in 2014 to a cold spot in 2015 (Figures4^10^).

**Figure 5.**
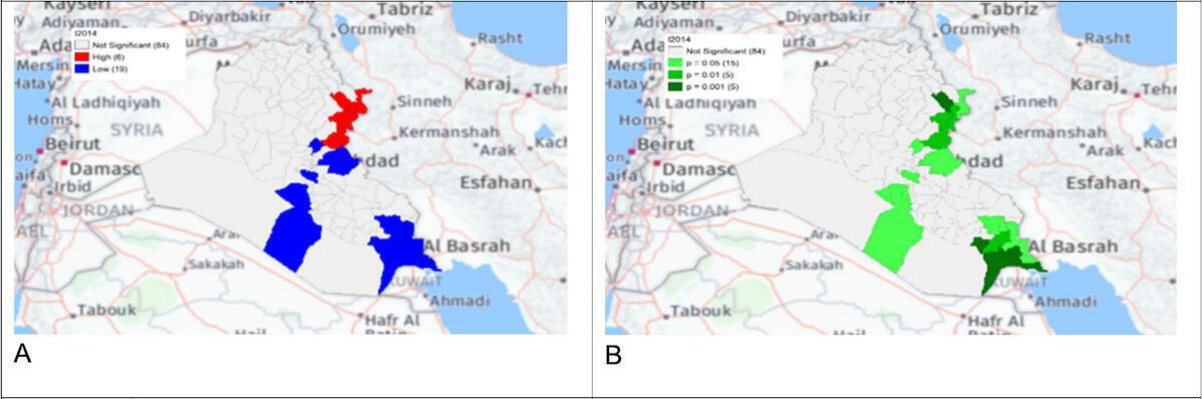
Gi* Cluster (A) and Gi* Significance (B) map of human brucellosis cases in Iraq in 2014.

**Figure 6.**
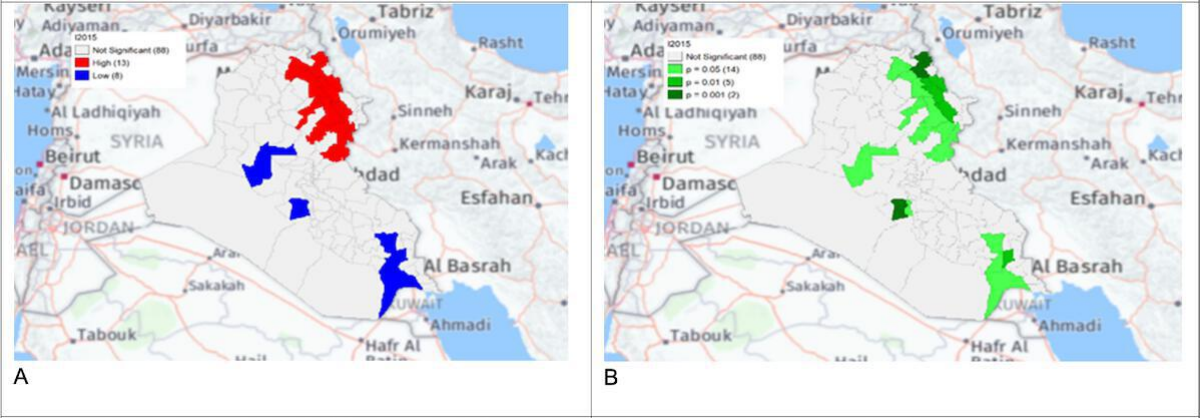
Gi* Cluster (A) and Gi* Significance (B) map of human brucellosis cases in Iraq in 2015.

**Figure 7.**
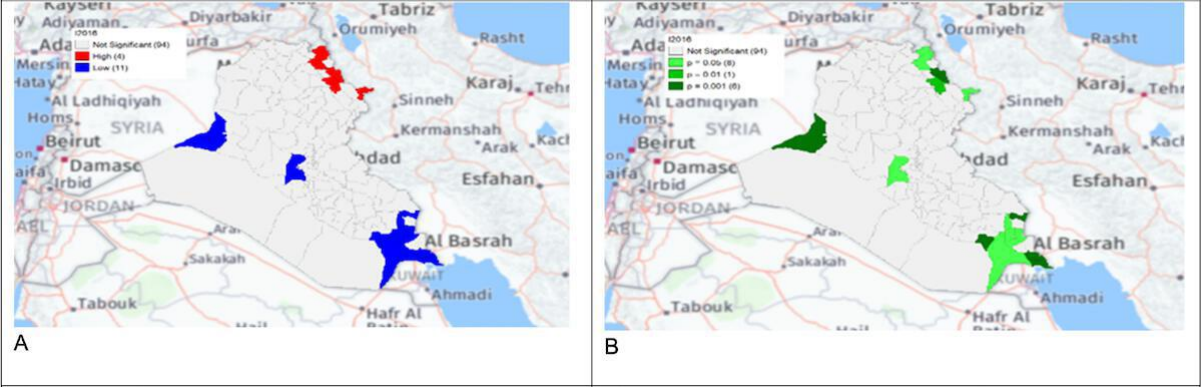
Gi* Cluster (A) and Gi* Significance (B) map of human brucellosis cases in Iraq in 2016.

**Figure 8.**
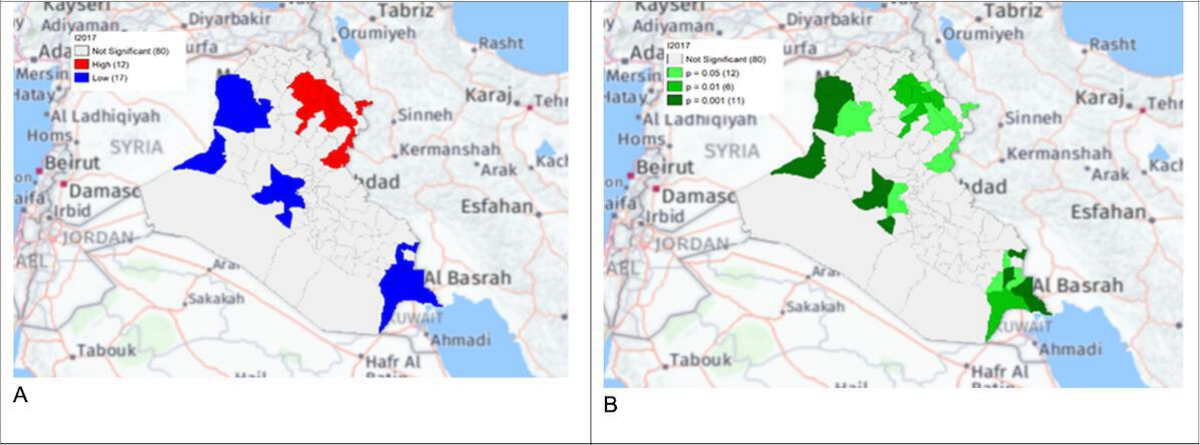
Gi* Cluster (A) and Gi* Significance (B) map of human brucellosis cases in Iraq in 2017.

**Figure 9.**
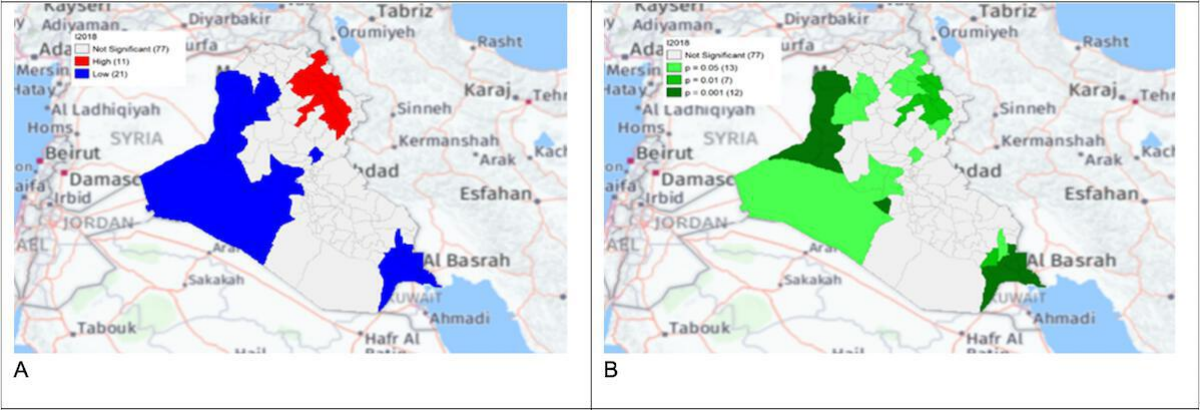
Gi* Cluster (A) and Gi* Significance (B) map of human brucellosis cases in Iraq in 2018.

**Figure 10.**
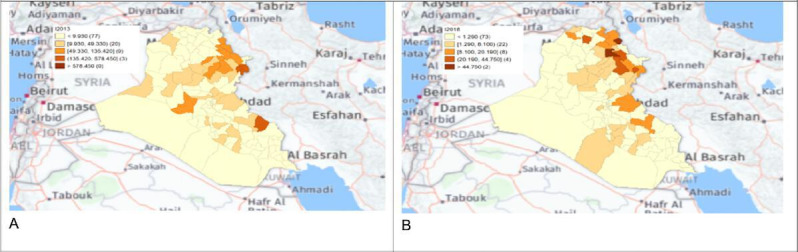
Spatial distribution maps of human brucellosis incidence rate in 2013 (A) and 2018 (B).

## Discussion

### Principal Findings

Analyzing the spatiotemporal distribution of human brucellosis is important in understanding the epidemiology of the disease in the country and helps forecast the future situation. There has been a decline in human brucellosis cases since 2012, which may be due to veterinary protective and control measures through vaccinating livestock that have been implemented since 2007. However, vaccination rounds were disrupted during Islamic State in Iraq and the Levant (ISIL) events that negatively affected the control program [11,12]. Nevertheless, animal health campaigns were relaunched soon after liberation in 2017, with several vaccination rounds having been completed since then [13].

The number of female individuals who tested positive for brucellosis has been constantly higher than the number of male individuals, with increased reporting frequency since 2015. This can be due to increased exposure of female individuals to infected animals and animal products through housekeeping and farming activities [11]. This contradicts what has been found in research from Germany [1], Italy [14], and Iran [8]. Therefore, the potential differences should be investigated further to detect whether the difference is due to different proportions of male individuals in different countries or biological differences that enhance contracting brucellosis. Nearly two-thirds (30,977/50,621, 61.19%) of the patients were between 15 and 45 years old, which can negatively affect the economic status of the patients and their families and the quality of life for the patients [9]. People in this age group may consume more locally made dairy products due to a lack of knowledge regarding the importance of pasteurized milk and milk products. However, this age category was very broad and could have been classified into 2-3 age groups to detect the most commonly affected age group [15-18].

Most human brucellosis cases were in the northern provinces during the first period (2007‐2012), especially in Salah Al-Din, Sulaymaniyah, Nineveh, Erbil, Kirkuk, and Dahuk. Northern provinces share an extensive border with Iran, Turkey, and Syria and direct borders with other Iraq provinces, which, in turn, share borders with Jordan, Saudi Arabia, and Kuwait [16,17]. Sheep and goat husbandry has been practiced in the northern region of Iraq since the earliest times due to the nature of these provinces, which is characterized by broad grass-covered terrain, undulating hills steep, and craggy mountains well suited to sheep and goat grazing [19]. Livestock and its products dominate northern Iraq’s economy and represents an important food resource for its inhabitants [5]. Therefore, the high clustering of brucellosis in this area could be due to area grazing strategy, animal population density, owner’s ignorance of the hazards of the disease, unhygienic disposal of infected animals as well as aborted fetuses or placental membranes, and uncontrolled movement of diseased animals [18]. Because there is no curative medical therapy for animal brucellosis, the infected animals should be slaughtered and disposed of properly. However, these animals are expensive in Iraq; therefore, farmers firmly refuse to slaughter and dispose them. As a result, the infection will persist through transmission from the infected animals to their offspring by breastfeeding. Likewise, humans may consume this infected, unpasteurized milk, resulting in infection and areas endemic with brucellosis in animals and humans [15]. One potential control method in this case is the isolation of the infected animal from healthy animals and milk pasteurization to prevent animal-to-animal and animal-to-human transmissions. Preventive measures such as health education activities should be performed in high-risk areas. Adopting the Quarantine-Slaughter-Immunization strategy and One Health Approach is crucial in controlling the disease. This can be achieved through multisectoral coordination and coordination with neighboring countries in the control programs.

The seasonal variation in the occurrence of human brucellosis in Iraq, that is, the decline in the number of human brucellosis cases in winter and the rise in spring and summer, could be explained by high exposure to the disease during spring and summer when the deliveries of animals, increased milk production, and contamination occur [19,20].

The human brucellosis cases were clustered in the north and northeastern regions of Iraq, mainly in areas in the vicinity of the Zagros mountains, which contain dense oak forests and fertile soil with a high density of sheep and goats; the main economic activity in the area is animal farming. The Zagros Mountains are also the route of seasonal migration for nomads [19]. Human-to-human transmission is rare and clusters of human cases are most likely a result of animal processing, more intensive agricultural production zones, and similar sociocultural practices [14].

On the contrary, the low cluster areas may be due to having economic superiority, standardized industrial manufacturing for cow- or sheep-related products, good sanitary habits, awareness of human brucellosis, and easy access to immediate treatment after infection [4,21]. In addition, several changes in the surveillance case definition, the diagnostic methods, and the reporting of human brucellosis that occurred during the second period of the study could have also contributed to the low numbers reported compared to the first period. For example, the primary diagnostic method during the first period was the Rose-Bengal test, which has variable sensitivity and specificity based on the exposure history, stage of infection, and prior infection history. In contrast, the diagnostic test used during the second period was ELISA for immunoglobulin M, immunoglobulin G, and culture. Although all suspected cases are initially reported to the Surveillance Section, their final classification as suspected or confirmed may vary by governorates based on their testing and interpretation capacity (Surveillance Section, CDC, Public Health Directorate, MoH in Iraq; unpublished data; 2024). As another example, the data were collected in an aggregate format based on sex, age group, and province in the first period of the study. In contrast, the data were collected in a case-based format during the second period of the study (Surveillance Section, CDC, Public Health Directorate, MoH in Iraq; unpublished data; 2024).

This study has several strengths. First, this is the first study to include data across 12 years in a detailed spatiotemporal analysis. The consistency of the epidemiological characteristics across 12 years can lead to public health interventions in areas where effort should be spent. That is, due to the fact that the infection is consistently more frequent among female individuals, health promotion activities may be directed to design educational materials on dealing with animals and their products and delivering them using personal protective measures that target female individuals working in the animal husbandry industry. The second strength is the use of an innovative statistical approach to detect whether the clustering of cases is significant or random, in addition to follow-up of the progress of clusters over 7 years, which clearly showed a continued increase in the north and northeastern areas and a continuous decline in the southern areas.

This study also has limitations. Data regarding important risk factors of human brucellosis, such as occupation, rural or urban areas, comorbidities, and treatment protocol, were lacking. Had this information been available, it would have facilitated a better understanding of the epidemiology and characteristics of human brucellosis in Iraq (Surveillance Section, CDC, Public Health Directorate, MoH in Iraq; unpublished data; 2024).

### Conclusions

Despite the declining incidence of human brucellosis in Iraq from 2013 to 2018, human brucellosis is still endemic and constitutes a public health problem. Most cases were reported in the summer and spring seasons among female individuals and those aged 15‐45 years. Human brucellosis cases presented significant spatial clustering in northern and northeastern areas.

## Abbreviations

CDC: Communicable Diseases Control Center
ELISA: enzyme-linked immunosorbent assay
ISIL: Islamic State in Iraq and the Levant
MoH: Ministry of Health
