# Correction: Human Brucellosis in Iraq: Spatiotemporal Data Analysis From 2007-2018

**DOI:** 10.2196/64958

**Published:** 2024-08-09

**Authors:** Ali Hazim Mustafa, Hanan Abdulghafoor Khaleel, Faris Lami

**Affiliations:** 1 Department of Inspection, Ministry of Health, Baghdad, Iraq; 2 Surveillance Section, Communicable Diseases Control Center, Public Health Directorate, Ministry of Health, Baghdad, Iraq; 3 College of Medicine, University of Baghdad, Baghdad, Iraq

In “Human Brucellosis in Iraq: Spatiotemporal Data Analysis From 2007-2018” (JMIRx Med 2024;5:e54611) the authors noted one error.

The originally published manuscript was missing some figures. The following figures have been added to the published paper (Figures4^10^):

**Figure 4.**
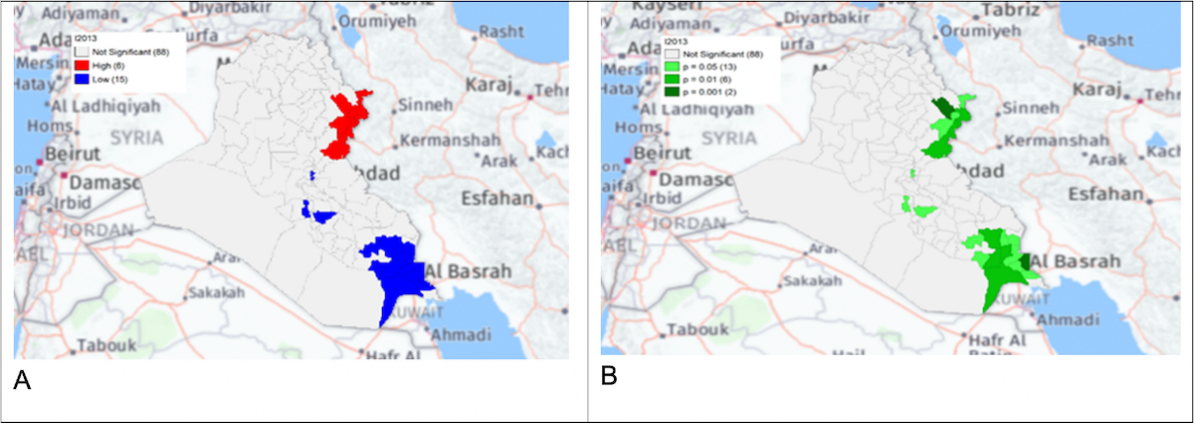
Gi* Cluster (A) and Gi* Significance (B) map of human brucellosis cases in Iraq in 2013.

**Figure 5.**
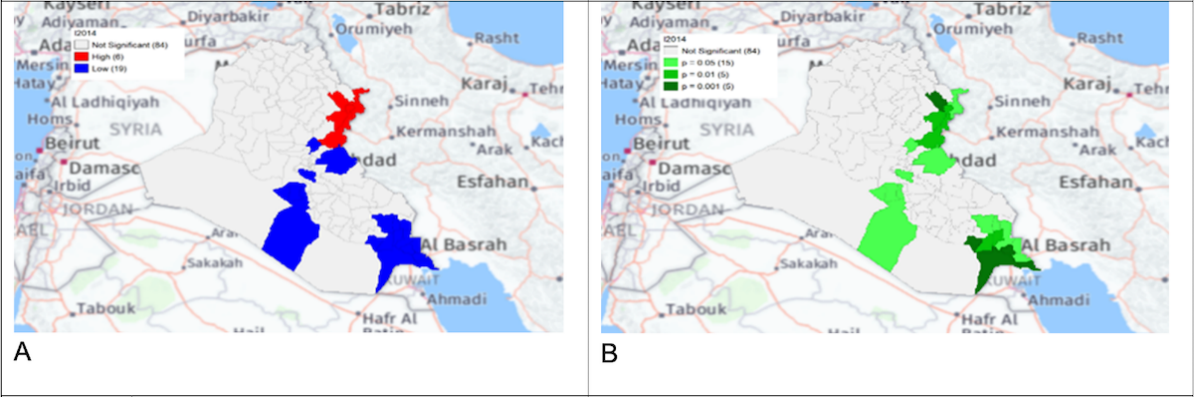
Gi* Cluster (A) and Gi* Significance (B) map of human brucellosis cases in Iraq in 2014.

**Figure 6.**
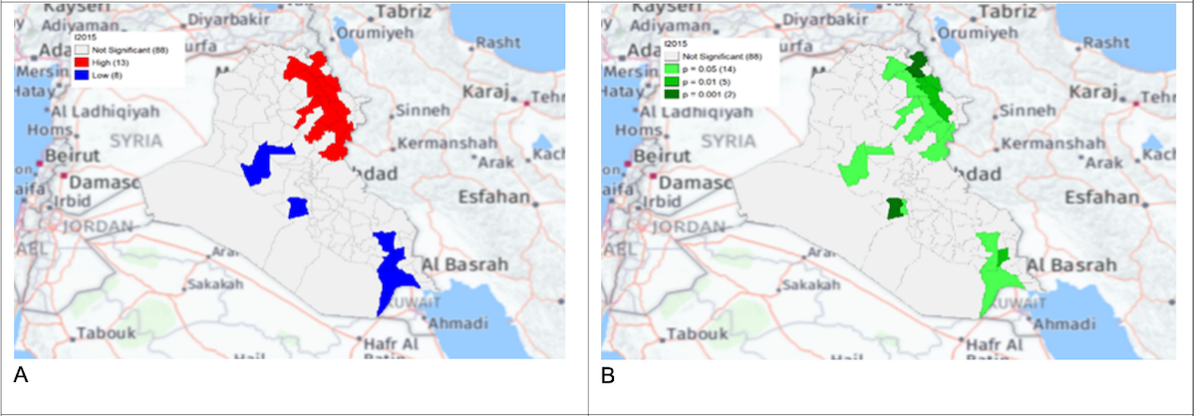
Gi* Cluster (A) and Gi* Significance (B) map of human brucellosis cases in Iraq in 2015.

**Figure 7.**
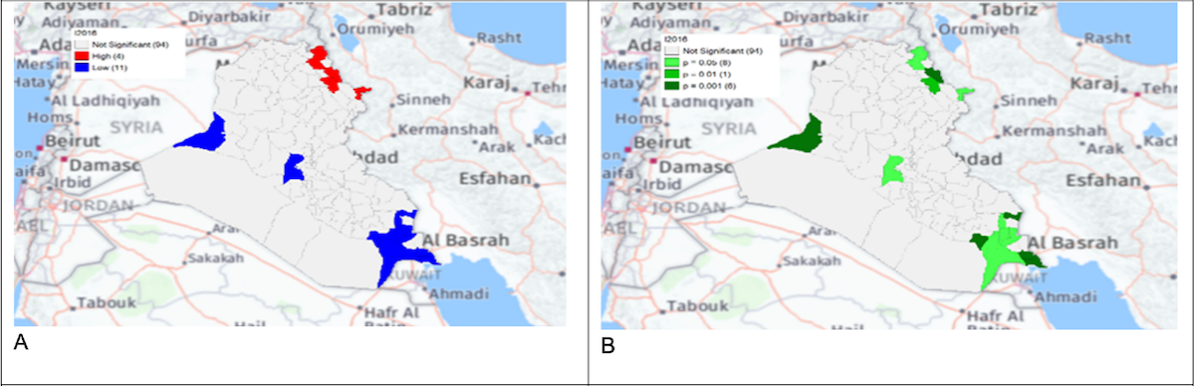
Gi* Cluster (A) and Gi* Significance (B) map of human brucellosis cases in Iraq in 2016.

**Figure 8.**
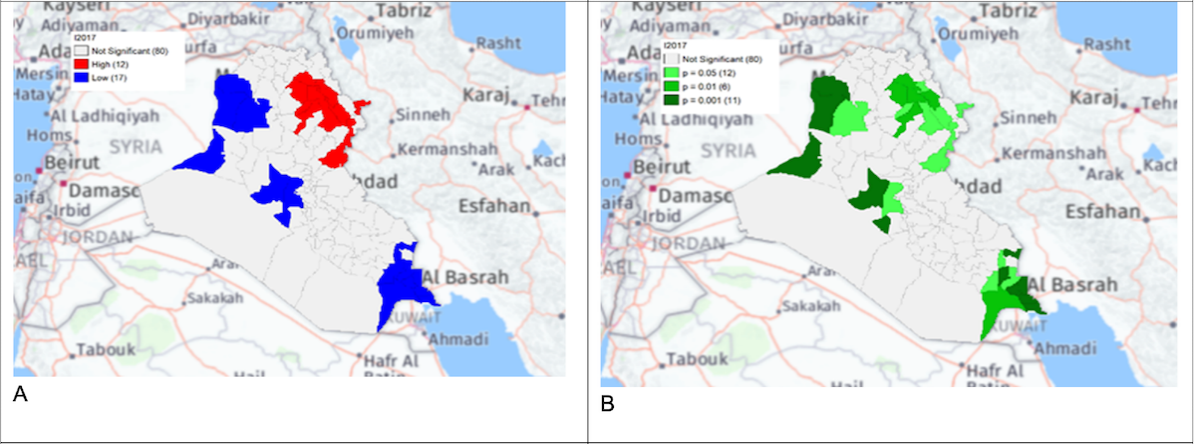
Gi* Cluster (A) and Gi* Significance (B) map of human brucellosis cases in Iraq in 2017.

**Figure 9.**
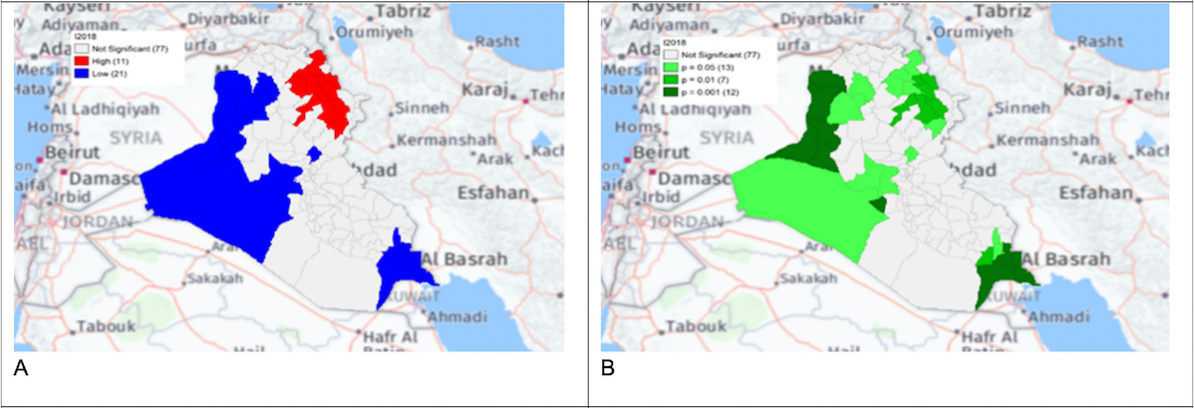
Gi* Cluster (A) and Gi* Significance (B) map of human brucellosis cases in Iraq in 2018.

**Figure 10.**
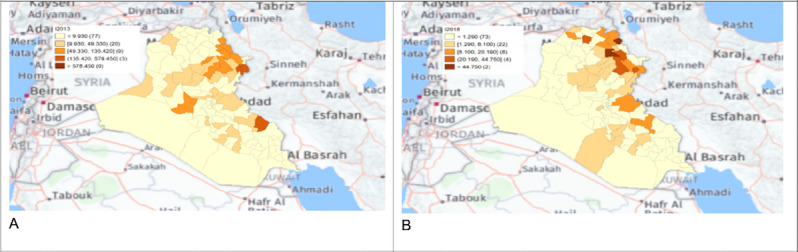
Spatial distribution maps of human brucellosis incidence rate in 2013 (A) and 2018 (B).

In addition, an in-text reference to these figures has been added to the final sentence of the Results section:

District Koisanjaq in Erbil shifted from a hot spot in 2014 to a cold spot in 2015 (Figures4^10^).

The correction will appear in the online version of the paper on the JMIR Publications website on August 9, 2024, together with the publication of this correction notice. Because this was made after submission to PubMed, PubMed Central, and other full-text repositories, the corrected article has also been resubmitted to those repositories.

